# The bioconcentration ability of heavy metal research for 50 kinds of rice under the same test conditions

**DOI:** 10.1007/s10661-016-5660-1

**Published:** 2016-11-16

**Authors:** Wen-Juan Xie, Lei Che, Guang-Yu Zhou, Li-Na Yang, Min-Yu Hu

**Affiliations:** 1 LuSong District Center for Disease Control and Prevention, Zhuzhou, 412000 China; 2 XiangYa School of Public Health, Central South University, Changsha, 410078 China

**Keywords:** Rice, Heavy metal, BCFs;lead, Cadmium

## Abstract

The aims of this experiment are to explore the accumulation of metal contamination of different varieties of rice planted in paddy fields and to provide a basis for the further research. The rice specimens were grown in and collected from a total area of 8.24 acres of rice planting fields where local farmers cultivated 50 different kinds of rice. The crops were grown using the methods of seedling, transplanting, fertilizing, and irrigation, under the guidance of professional and technical personnel. The 50 kinds of paddy rice contain 20 kinds of conventional rice, 15 kinds of two-line hybrid rice, 15 kinds of three-line hybrid rice, and the whole experiment lasted 100 days. To begin our analysis of the data, we first gathered 15 irrigation water samples respectively from the first day of the experiment. This was then followed by gathering water samples from the tillering stage, then the development stage, the solid phase, and finally, the last day of the experiment. On the first day and at the end of the experiment, we had respectively gathered 6 mud samples from the rice paddies, with a total 12 parts of it. In addition to this, by the end of the experiment, we had gathered 6 samples of rice spike from each type of the investigated rice, with a total 300 parts of it. These samples were then analyzed in the laboratory to detect the contents and amounts of lead, cadmium, chromium, arsenic, copper, calcium, fluoride, nitrogen, phosphorus, potassium in the samples, and the pH quality of the samples. The quality of irrigation water was evaluated according to irrigation water quality standards (GB 5084-2005); the rice paddy mud samples were detected and evaluated respectively according to farmland soil environment quality monitor technology standards (NY/T 395-2012) and the journal of environmental quality assessment standard of edible agricultural products (HJ 332-2006); the rice grains were detected and evaluated according to the limited food standards (GB 2762-2012); the bioaccumulation factors (BCFs) were adopted to evaluate the accumulation ability of metal contamination in rice. As a result, the test values of the irrigation water samples were within irrigation water quality standards. Only the content of cadmium was beyond the environmental quality assessment standard of edible agricultural products, by 0.07 mg/kg. The content of lead and cadmium in 50 different rice were 0.41 ± 0.01~0.49 ± 0.01 mg/kg and 0.22 ± 0.01~0.25 ± 0.01 mg/kg, respectively. The varietal differences were not statistically significant (*P*>0.05). Lead BCFs, cadmium BCFs, and chromium BCFs in 50 different kinds of rice had no statistical difference (*P*>0.05). For the content of lead, cadmium, chromium, inorganic arsenic and copper in the conventional rice samples, two-line hybrid rice samples, and three-line hybrid rice samples, there was no statistical difference (*P*>0.05). Lead BCFs, cadmium BCFs, chromium BCFs, arsenic BCFs, and copper BCFs also had no statistical difference (*P*>0.05). This means the content of cadmium and lead contaminant in the 50 kinds of rice exceeded food quality and limits. The content of cadmium of mud samples exceeded the assessment standard by 0.07 mg/kg, the content of cadmium, of the 50 kinds of rice, exceeded the limited food standard by 0.04 mg/kg. The content of lead in the paddy mud was within the limited value, but the content of lead exceeded the limited food standard by 0.24 mg/kg. For the lead BCFs, cadmium BCFs, and chromium BCFs of the 50 kinds of rice, there was no statistically significant difference. This was the same for lead BCFs, cadmium BCFs, chromium BCFs, arsenic BCFs, and copper BCFs during conventional rice, two-line hybrid rice, and three-line hybrid rice. For the above, the rice had a strong adsorption capacity of lead. The conclusions of this data lead us to not only implement measures of control but also to conduct research on the suitable levels of lead in edible agricultural products.

## Introduction

Heavy metal pollution of crops has become a global issue. The potential hazards and solutions are now a widespread concern of scholars (Li et al. 2004). Researches both domestically and abroad have been conducted on the heavy metal accumulator situation in different types of rice. Some studies have shown that, compared with conventional rice, hybrid rice has a higher absorption transport ability of Cd (Liu et al. 2004; Li et al. 2003; Zhong et al. 2001); however, contrary findings have also been reported (Li et al. 2013; Yan et al. 2006). In this study, through a farmland cultivation experiment in the same environment, we explored heavy metal enrichment situations in 50 different kinds of rice, providing a reference for further study about the safety of heavy metals detected in rice and providing additional data support for controlling said detected metals.

## Materials and methods

### Collecting samples

The 50 different kinds of rice are numbered sequentially, in which conventional rice is from 1 to 20, two-line hybrid rice is from 21 to 35, and three-line hybrid rice is from 36 to 50. Irrigation canal water samples were collected during the following stages: the first day of the experiment, the rice tillering stage, the development stage, the seed setting stage, and the end of the experiment, respectively, with a total of 15 parts with each sample measured at around 500 mL. On the first day of the experiment and at the end of the experiment, samples of six parts each day were collected, with 0–20 cm of topsoil mud, using the plum-sampling method, where each sample contained 1 kg of each forming a total of 300 copies at about 800 g per sample.

### Preparing samples

Irrigation canals’ water is processed by agricultural water environment quality monitoring technology (NY/T 396-2000). Soil samples are processed by farmland soil environment monitoring technology (NY/T 395-2012); soil samples are dried naturally at room temperature and crushed with sticks, debris is removed, then filtered by a diameter 0.25 mm (100 mesh) sieve, mixed the samples fully, saved in a plastic bottle, and set aside. After drying the rice samples in the sun, the small milling machines divide up the grains into rice and bran, with the ratio of rice at 72%. Then, the rice is crushed with a stamp smash and filtered by a diameter 0.25 mm (100 mesh) sieve, and then placed into plastic bottles and set aside.

### Laboratory test methods

The lead and Cd in the irrigation canal water, the soil and the rice, and the chrome in the soil and rice, were detected by the graphite furnace atomic absorption method, while chrome in the irrigation canal water was detected by the potassium permanganate oxidation-II benzene carbon n II hydrazine point light photometric method; the total arsenic in irrigation canals’ water and soil were detected by the II b base II sulfur generation amino carboxylic acid silver point light photometric method; inorganic arsenic in rice was detected by the liquid phase color spectrum-inductance coupled plasma quality spectrum method; potassium, copper, and the calcium in irrigation canals’ water, soil, and rice were detected by the flame atomic absorption point light photometric method. The content of fluorine was detected by the ion selective electrode method, while the content of phosphorus was detected by vanadium-ammonium molybdate spectrophotometry; the total nitrogen content in the canal water was detected by alkaline potassium persulfate digestion-UV-spectrophotometric method; the nitrogen content in the soil and grains were detected by the Kjeldahl method; the pH of the irrigation water was tested by agro-technical specification for water quality monitoring; the pH of the soil was tested by edible agricultural products of environment quality monitoring technology.

### Evaluation criteria

The irrigation water was evaluated by irrigation water quality standards (GB 5084-2005), while mud samples were evaluated by edible farm products in paddy fields of environment quality evaluation criteria (HJ 332-2006). All kinds of rice were evaluated by the limits of pollutants in food (GB 2762-2012). The bioconcentration ability of rice to heavy metals was evaluated by BCFs. The bigger the BCFs are, the stronger the rice capacity of absorption and accumulation to heavy metals will be. The BCFs were calculated by the following equation:

$$ \mathrm{BCFs}=\frac{\mathrm{heavy}\ \mathrm{metal}\ \mathrm{content}\ \mathrm{in}\ \mathrm{rice}}{\mathrm{heavy}\ \mathrm{metal}\ \mathrm{content}\ \mathrm{in}\ \mathrm{soil}} $$

### Statistical analysis

We adopted SPSS 18.0 to analyze data, where mean ± standard deviation ($\overline{X}\pm SD$) refers to the average and dispersed degree of the data. According to the normality and *f* test results, we compared the means of the two groups using the independent sample *t* test, but only if the data obeyed the normal distribution and equal variance. We also compared the means of multiple sets of samples using the single factor analysis of variance, but only if the data obeyed the normal distribution and equal variance, as set by the SNK-q test. The Kruskal-Wallis *H* test was used to compare the means of the groups which their data is not in normal distribution or equal variances, followed by post hoc of variance analysis of rank transformation technique combined with completely random design. The pre- and post-experiment data were compared with a paired sample *t* test. Significance level α is 0.05.

## Results

### General information

During the experiment, we have observed rice growing on an irregular basis. The stem length, spike length, and full status of the grain were found to have no significant differences. The experimental period of this study covered 100 days.

### Heavy metal concentrations of irrigation water

The test results of irrigation water are shown in Table 1.

**Table 1:** Irrigation water test results (*n* = 3, mg/kg,$\overline{X}\pm SD$)

	Limit standard	Day 1	Tillering stage	Growth period	Grain filling stage	Last day
pH	5.5~8.5	7.40 ± 0.10^a^	7.50 ± 0.10^a^	7.40 ± 0.05^a^	7.30 ± 0.10^a^	7.20 ± 0.20^a^
Lead	≤0.2	0.014 ± 0.002^a^	0.015 ± 0.001^a^	0.015 ± 0.002^a^	0.015 ± 0.002^a^	0.015 ± 0.002^a^
Cadmium	≤0.01	0.00019 ± 0.00001^a^	0.00020 ± 0.00001^a^	0.00018 ± 0.00001^a^	0.00019 ± 0.00001^a^	0.00020 ± 0.00001^a^
Chromium	≤0.1	0.080 ± 0.0024^a^	0.077 ± 0.0026^a^	0.079 ± 0.0025^a^	0.080 ± 0.0022^a^	0.079 ± 0.0026^a^
Arsenic	≤0.05	0.020 ± 0.005^a^	0.020 ± 0.004^a^	0.019 ± 0.006^a^	0.021 ± 0.005^a^	0.020 ± 0.004^a^
Copper	≤0.5	0.104 ± 0.043^a^	0.117 ± 0.014^a^	0.114 ± 0.039^a^	0.115 ± 0.012^a^	0.112 ± 0.032^a^
Fluorine	≤2	0.207 ± 0.003^a^	0.210 ± 0.002^a^	0.211 ± 0.003^a^	0.212 ± 0.002^a^	0.209 ± 0.003^a^
Nitrogen	–	0.092 ± 0.004^a^	0.108 ± 0.003^a^	0.116 ± 0.002^a^	0.096 ± 0.005^a^	0.102 ± 0.004^a^
Phosphorus	–	0.280 ± 0.086^a^	0.309 ± 0.043^a^	0.299 ± 0.065^a^	0.326 ± 0.057^a^	0.303 ± 0.044^a^
Potassium	–	4.199 ± 0.133^a^	4.212 ± 0.093^a^	4.177 ± 0.098^a^	4.211 ± 0.102^a^	4.200 ± 0.109^a^
Calcium	–	0.438 ± 0.034^a^	0.464 ± 0.040^a^	0.444 ± 0.033^a^	0.445 ± 0.053^a^	0.448 ± 0.044^a^

The data with different superscript letters within the same column means significant difference, *P*<0.05, with the same superscript letter means no significant difference, *P*>0.05. *H*_铅_ = 0. 431, *P* = 0.732, *F*_镉_ = 0.324, *P* = 0.808, *H*_铬_ = 0.150, *P* = 0.929;*F*_砷_ = 0.132, *P* = 0.940, *F*_铜_ = 0.238, *P* = 0.870, *H*_氟_ = 1.616, *P* = 0.199, *H*_氮_ = 1.173, *P* = 0.331, *F*_磷_ = 1.429, *P* = 0.247, *F*_钾_ = 0.323, *P* = 0.808, *F*_钙_ = 0.720, *P* = 0.545

Table 1 shows pH, lead, cadmium, chromium, arsenic, and copper content in irrigation water are within the limit values of irrigation water quality standards, and there is no difference in the pH levels of the irrigation water during different time periods, where the lead, cadmium, chromium, arsenic, and copper content were all around *P* > 0.05.

### Heavy metal concentrations of soil samples in rice field

Table 2 illustrates the test results of the experiment on soil samples in the rice fields on the first day and last day.

**Table 2:** Test results of the soil samples on the first day and the last day of the experiment (*n* = 6, $\overline{X}\pm SD$)

	Limit standard	Day 1	Last day	*t*	*P*
pH	Null	7.00 ± 0.18	6.80 ± 0.09	3.31	0.12
Lead(mg/kg)	≤80 mg/kg(pH 6.5–7.5)	39.56 ± 2.16	38.15 ± 0.27	0.60	0.57
Cadmium (mg/kg)	≤0.30 mg/kg(pH 6.5–7.5)	0.37 ± 0.01^#^	0.33 ± 0.01^#^	9.27	0.10
Chromium (mg/kg)	≤300 mg/kg(pH 6.5–7.5)	39.10 ± 1.13	35.26 ± 0.23	9.80	0.06
Arsenic (mg/kg)	≤25 mg/kg(pH 6.5–7.5)	1.22 ± 0.03	0.83 ± 0.01*	37.90	0.04
Copper (mg/kg)	≤100 mg/kg(pH 6.5–7.5)	72.38 ± 2.39	30.16 ± 2.15*	30.72	0.00
Fluorine (mg/kg)	Null	216.06 ± 4.60	204.72 ± 3.12	4.45	0.15
Nitrogen (mg/kg)	Null	1970.34 ± 0.07	1610.23 ± 10.09	2.90	0.21
Phosphorus (mg/kg)	Null	825.33 ± 41.16	755.02 ± 2.71	1.34	0.14
Potassium (mg/kg)	Null	18,804.00 ± 382.18	14,390.83 ± 278.33	9.67	0.11
Calcium (mg/kg)	Null	8254.67 ± 37.57	8024.17 ± 1.47	15.35	0.10

Data with signal * means it is beyond the limited standards of the state and represents statistically significant difference on the first day and last day of the experiment (*P* < 0.05)

Table 2 demonstrates the cadmium level of the soil samples in the rice paddy field has exceeded the limit which set by the environmental quality of edible agricultural soils at the first day of the experiment. At the end of the experiment, the pH of the soil sample from the rice paddy was discovered to be 6.80 ± 0.09. The arsenic and copper contents were both reduced compared to the levels recorded on the first day before planting, (*P* < 0.05).

### Heavy metal concentrations of rice

The test results of 50 kinds of rice are shown in Table 3.

**Table 3:** illustrates lead, cadmium, and chromium contents have no noticeable differences among the various species of rice (*P* > 0.05). The inorganic arsenic of no. 40, 47, and 48 varieties are lower than that of the other varieties, which has a statistically significant difference of *P* < 0.05. The copper content of the no. 20 varieties is relatively lower than that of the other varieties, with a statistically significant difference of *P* < 0.05. The copper contents of the no. 42 and 43 varieties are higher than that of the other varieties, with a statistically significant difference of *P* < 0.05

	Lead (mg/kg)	Cadmium (mg/kg)	Chromium (mg/kg)	Inorganic arsenic (mg/kg)	Fluorine (mg/kg)	Copper (mg/kg)	Crude protein (g/100 g)	Phosphorus (mg/kg)	Potassium (mg/kg)	Calcium (mg/kg)
	≤0.2	≤0.2	≤1.0	≤0.2	–	–	–	–	–	–
1	0.47 ± 0.01^a#^	0.22 ± 0.01^a#^	0.52 ± 0.01^a^	0.09 ± 0.01^a^	0.92 ± 0.01^a^	4.53 ± 0.26^a^	9.24 ± 0.03^a^	1.60 ± 0.01^a^	1162.75 ± 17.23^a^	61.36 ± 4.81^a^
2	0.47 ± 0.01^a#^	0.22 ± 0.01^a#^	0.54 ± 0.01^a^	0.09 ± 0.01^a^	0.94 ± 0.01^a^	5.86 ± 0.37^a^	9.42 ± 0.03^a^	1.59 ± 0.01^a^	915.33 ± 10.40^a^	52.54 ± 2.61^a^
3	0.49 ± 0.01^a#^	0.22 ± 0.01^a#^	0.57 ± 0.01^a^	0.10 ± 0.01^a^	0.86 ± 0.01^a^	5.42 ± 0.19^a^	8.63 ± 0.03^a^	1.61 ± 0.01^a^	1151.29 ± 21.79^a^	52.80 ± 2.64^a^
4	0.46 ± 0.01^a#^	0.22 ± 0.01^a#^	0.53 ± 0.01^a^	0.09 ± 0.01^a^	0.84 ± 0.01^a^	5.33 ± 0.16^a^	8.37 ± 0.02^a^	1.65 ± 0.01^a^	928.14 ± 11.73^a^	56.20 ± 3.56^a^
5	0.45 ± 0.01^a#^	0.22 ± 0.01^a#^	0.55 ± 0.01^a^	0.09 ± 0.01^a^	0.85 ± 0.01^a^	5.37 ± 0.20^a^	8.55 ± 0.01^a^	1.61 ± 0.01^a^	1030.73 ± 83.11^a^	44.21 ± 7.08^a^
6	0.45 ± 0.01^a#^	0.22 ± 0.01^a#^	0.54 ± 0.01^a^	0.09 ± 0.01^a^	0.92 ± 0.01^a^	4.73 ± 0.10^a^	9.24 ± 0.01^a^	1.60 ± 0.01^a^	910.68 ± 26.14^a^	50.02 ± 1.78^a^
7	0.45 ± 0.01^a#^	0.22 ± 0.01^a#^	0.53 ± 0.01^a^	0.09 ± 0.01^a^	0.82 ± 0.01^a^	6.82 ± 0.38^a^	8.21 ± 0.02^a^	1.59 ± 0.01^a^	1028.36 ± 25.99^a^	53.94 ± 4.21^a^
8	0.47 ± 0.01^a#^	0.22 ± 0.01^a#^	0.52 ± 0.01^a^	0.10 ± 0.01^a^	0.89 ± 0.01^a^	5.26 ± 0.42^a^	8.85 ± 0.01^a^	1.59 ± 0.01^a^	965.13 ± 31.02^a^	57.00 ± 2.47^a^
9	0.48 ± 0.01^a#^	0.22 ± 0.01^a#^	0.53 ± 0.01^a^	0.09 ± 0.01^a^	0.92 ± 0.01^a^	3.41 ± 0.21^a^	9.23 ± 0.01^a^	1.61 ± 0.01^a^	1129.58 ± 29.71^a^	52.41 ± 4.83^a^
10	0.46 ± 0.01^a#^	0.22 ± 0.01^a#^	0.51 ± 0.01^a^	0.09 ± 0.01^a^	0.92 ± 0.01^a^	5.45 ± 0.32^a^	9.33 ± 0.02^a^	1.58 ± 0.01^a^	965.44 ± 16.65^a^	54.31 ± 2.50^a^
11	0.48 ± 0.01^a#^	0.22 ± 0.01^a#^	0.55 ± 0.01^a^	0.09 ± 0.01^a^	0.93 ± 0.01^a^	4.79 ± 0.09^a^	9.62 ± 0.02^a^	1.62 ± 0.01^a^	1014.28 ± 52.36^a^	57.30 ± 2.90^a^
12	0.48 ± 0.01^a#^	0.22 ± 0.01^a#^	0.55 ± 0.01^a^	0.09 ± 0.01^a^	0.96 ± 0.01^a^	5.47 ± 0.27^a^	8.63 ± 0.02^a^	1.61 ± 0.01^a^	1267.20 ± 27.59^a^	58.26 ± 3.61^a^
13	0.47 ± 0.01^a#^	0.22 ± 0.01^a#^	0.55 ± 0.01^a^	0.10 ± 0.01^a^	0.86 ± 0.01^a^	6.09 ± 0.19^a^	9.35 ± 0.02^a^	1.60 ± 0.01^a^	950.48 ± 28.03^a^	58.55 ± 5.34^a^
14	0.45 ± 0.01^a#^	0.22 ± 0.01^a#^	0.52 ± 0.01^a^	0.09 ± 0.01^a^	0.94 ± 0.01^a^	5.68 ± 0.39^a^	7.74 ± 0.01^a^	1.60 ± 0.01^a^	939.64 ± 46.94^a^	54.07 ± 2.50^a^
15	0.47 ± 0.01^a#^	0.22 ± 0.01^a#^	0.54 ± 0.01^a^	0.09 ± 0.01^a^	0.77 ± 0.01^a^	6.14 ± 0.09^a^	7.40 ± 0.01^a^	1.59 ± 0.01^a^	988.16 ± 15.13^a^	57.64 ± 2.01^a^
16	0.46 ± 0.01^a#^	0.22 ± 0.01^a#^	0.56 ± 0.01^a^	0.09 ± 0.01^a^	0.74 ± 0.01^a^	7.69 ± 0.24^a^	8.66 ± 0.02^a^	1.62 ± 0.01^a^	967.07 ± 44.14^a^	59.53 ± 3.56^a^
17	0.47 ± 0.01^a#^	0.22 ± 0.01^a#^	0.52 ± 0.01^a^	0.10 ± 0.01^a^	0.87 ± 0.01^a^	6.94 ± 0.29^a^	8.77 ± 0.02^a^	1.60 ± 0.01^a^	1156.77 ± 23.22^a^	53.05 ± 3.89^a^
18	0.45 ± 0.01^a#^	0.22 ± 0.01^a#^	0.53 ± 0.01^a^	0.09 ± 0.01^a^	0.88 ± 0.01^a^	3.60 ± 0.16^a^	8.67 ± 0.02^a^	1.59 ± 0.01^a^	1035.57 ± 28.22^a^	54.40 ± 4.26^a^
19	0.47 ± 0.01^a#^	0.24 ± 0.01^a#^	0.54 ± 0.01^a^	0.14 ± 0.01^a^	0.87 ± 0.01^a^	4.98 ± 0.08^a^	8.86 ± 0.02^a^	1.95 ± 0.01^a^	1086.98 ± 29.99^a^	56.02 ± 1.62^a^
20	0.42 ± 0.01^a#^	0.24 ± 0.01^a#^	0.58 ± 0.01^a^	0.14 ± 0.01^a^	0.89 ± 0.01^a^	2.48 ± 0.12^b^	8.66 ± 0.02^a^	1.46 ± 0.01^a^	992.95 ± 91.86^a^	60.67 ± 2.04^a^
21	0.42 ± 0.01^a#^	0.24 ± 0.01^a#^	0.59 ± 0.01^a^	0.14 ± 0.01^a^	0.87 ± 0.01^a^	5.02 ± 0.09^a^	8.51 ± 0.02^a^	1.65 ± 0.01^a^	994.44 ± 21.02^a^	58.41 ± 1.89^a^
22	0.42 ± 0.01^a#^	0.22 ± 0.01^a#^	0.58 ± 0.01^a^	0.09 ± 0.01^a^	0.85 ± 0.01^a^	4.21 ± 0.09^a^	8.77 ± 0.02^a^	1.60 ± 0.01^a^	1079.82 ± 19.68^a^	52.73 ± 3.29^a^
23	0.46 ± 0.01^a#^	0.22 ± 0.01^a#^	0.52 ± 0.01^a^	0.09 ± 0.01^a^	0.88 ± 0.01^a^	5.65 ± 0.07^a^	9.45 ± 0.01^a^	1.61 ± 0.01^a^	929.84 ± 9.22^a^	59.39 ± 2.87^a^
24	0.45 ± 0.01^a#^	0.24 ± 0.01^a#^	0.53 ± 0.01^a^	0.16 ± 0.01^a^	0.94 ± 0.01^a^	4.19 ± 0.03^a^	8.36 ± 0.01^a^	1.65 ± 0.01^a^	949.73 ± 24.77^a^	58.99 ± 2.69^a^
25	0.42 ± 0.01^a#^	0.25 ± 0.01^a#^	0.59 ± 0.01^a^	0.15 ± 0.01^a^	0.84 ± 0.01^a^	4.81 ± 0.04^a^	8.29 ± 0.02^a^	1.66 ± 0.01^a^	945.08 ± 119.74^a^	58.39 ± 4.42^a^
26	0.42 ± 0.01^a#^	0.25 ± 0.01^a#^	0.60 ± 0.01^a^	0.15 ± 0.01^a^	0.84 ± 0.01^a^	4.85 ± 0.06^a^	8.38 ± 0.01^a^	1.59 ± 0.01^a^	918.17 ± 22.97^a^	54.59 ± 4.27^a^
27	0.42 ± 0.01^a#^	0.25 ± 0.01^a#^	0.58 ± 0.01^a^	0.09 ± 0.01^a^	0.87 ± 0.01^a^	4.52 ± 0.08^a^	8.66 ± 0.01^a^	1.60 ± 0.01^a^	907.53 ± 60.26^a^	56.95 ± 2.85^a^
28	0.41 ± 0.01^a#^	0.25 ± 0.01^a#^	0.59 ± 0.01^a^	0.13 ± 0.01^a^	0.85 ± 0.01^a^	4.50 ± 0.04^a^	8.45 ± 0.01^a^	1.66 ± 0.01^a^	951.36 ± 21.15^a^	55.20 ± 4.19^a^
29	0.45 ± 0.01^a#^	0.25 ± 0.01^a#^	0.57 ± 0.01^a^	0.09 ± 0.01^a^	0.85 ± 0.01^a^	7.16 ± 0.06^a^	8.48 ± 0.02^a^	1.63 ± 0.01^a^	991.98 ± 81.14^a^	56.12 ± 3.13^a^
30	0.41 ± 0.01^a#^	0.24 ± 0.01^a#^	0.60 ± 0.01^a^	0.09 ± 0.01^a^	0.84 ± 0.01^a^	5.34 ± 0.06^a^	8.38 ± 0.01^a^	1.56 ± 0.01^a^	940.15 ± 15.62^a^	52.86 ± 5.27^a^
31	0.43 ± 0.01^a#^	0.25 ± 0.01^a#^	0.59 ± 0.01^a^	0.11 ± 0.01^a^	0.85 ± 0.01^a^	5.69 ± 0.05^a^	8.45 ± 0.01^a^	1.64 ± 0.01^a^	903.61 ± 58.94^a^	53.95 ± 5.86^a^
32	0.42 ± 0.01^a#^	0.26 ± 0.01^a#^	0.57 ± 0.01^a^	0.12 ± 0.01^a^	0.83 ± 0.01^a^	7.21 ± 0.09^a^	8.33 ± 0.01^a^	1.65 ± 0.01^a^	946.97 ± 31.87^a^	59.35 ± 0.80^a^
33	0.44 ± 0.01^a#^	0.25 ± 0.01^a#^	0.59 ± 0.01^a^	0.15 ± 0.01^a^	0.84 ± 0.01^a^	3.84 ± 0.12^a^	8.41 ± 0.01^a^	1.72 ± 0.01^a^	896.12 ± 80.20^a^	57.90 ± 2.80^a^
34	0.43 ± 0.01^a#^	0.25 ± 0.01^a#^	0.59 ± 0.01^a^	0.15 ± 0.01^a^	0.74 ± 0.01^a^	5.85 ± 0.31^a^	7.40 ± 0.02^a^	1.73 ± 0.01^a^	907.83 ± 54.12^a^	57.21 ± 2.81^a^
35	0.43 ± 0.01^a#^	0.25 ± 0.01^a#^	0.62 ± 0.01^a^	0.12 ± 0.01^a^	0.78 ± 0.01^a^	3.40 ± 0.32^a^	7.84 ± 0.01^a^	1.40 ± 0.01^a^	944.02 ± 21.93^a^	60.80 ± 2.24^a^
36	0.42 ± 0.01^a#^	0.25 ± 0.01^a#^	0.60 ± 0.01^a^	0.14 ± 0.01^a^	0.73 ± 0.01^a^	3.63 ± 0.33^a^	7.34 ± 0.01^a^	1.53 ± 0.01^a^	946.70 ± 17.04^a^	57.58 ± 5.38^a^
37	0.42 ± 0.01^a#^	0.25 ± 0.01^a#^	0.60 ± 0.01^a^	0.14 ± 0.01^a^	0.84 ± 0.01^a^	5.92 ± 0.26^a^	8.42 ± 0.01^a^	1.33 ± 0.01^a^	904.38 ± 27.90^a^	55.75 ± 5.62^a^
38	0.42 ± 0.01^a#^	0.25 ± 0.01^a#^	0.57 ± 0.01^a^	0.10 ± 0.01^a^	0.84 ± 0.01^a^	3.50 ± 0.36^a^	8.39 ± 0.01^a^	1.33 ± 0.01^a^	920.83 ± 32.29^a^	43.63 ± 6.44^a^
39	0.41 ± 0.01^a#^	0.25 ± 0.01^a#^	0.57 ± 0.01^a^	0.11 ± 0.01^a^	0.84 ± 0.01^a^	3.52 ± 0.39^a^	8.45 ± 0.01^a^	1.34 ± 0.01^a^	947.99 ± 20.11^a^	49.36 ± 4.06^a^
40	0.42 ± 0.01^a#^	0.24 ± 0.01^a#^	0.58 ± 0.01^a^	0.05 ± 0.01^b^	0.85 ± 0.01^a^	5.27 ± 0.24^a^	8.51 ± 0.01^a^	1.98 ± 0.01^a^	993.26 ± 16.73^a^	52.07 ± 2.07^a^
41	0.42 ± 0.01^a#^	0.25 ± 0.01^a#^	0.58 ± 0.01^a^	0.09 ± 0.01^a^	0.86 ± 0.01^a^	3.62 ± 0.32^a^	8.61 ± 0.02^a^	1.53 ± 0.01^a^	1045.96 ± 24.62^a^	51.34 ± 4.28^a^
42	0.41 ± 0.01^a#^	0.25 ± 0.01^a#^	0.60 ± 0.01^a^	0.09 ± 0.01^a^	0.85 ± 0.01^a^	9.15 ± 0.05^c^	8.48 ± 0.01^a^	1.54 ± 0.01^a^	1161.78 ± 18.10^a^	49.07 ± 4.43^a^
43	0.42 ± 0.01^a#^	0.24 ± 0.01^a#^	0.59 ± 0.01^a^	0.08 ± 0.01^a^	0.84 ± 0.01^a^	8.13 ± 0.06^c^	8.36 ± 0.01^a^	1.52 ± 0.01^a^	846.65 ± 22.88^a^	47.69 ± 2.17^a^
44	0.44 ± 0.01^a#^	0.24 ± 0.01^a#^	0.60 ± 0.01^a^	0.08 ± 0.01^a^	0.83 ± 0.01^a^	4.48 ± 0.25^a^	8.27 ± 0.01^a^	1.54 ± 0.01^a^	918.58 ± 35.13^a^	48.08 ± 1.87^a^
45	0.42 ± 0.01^a#^	0.25 ± 0.01^a#^	0.58 ± 0.01^a^	0.09 ± 0.01^a^	0.84 ± 0.01^a^	4.21 ± 0.36^a^	8.36 ± 0.01^a^	1.56 ± 0.01^a^	908.55 ± 11.08^a^	48.26 ± 5.07^a^
46	0.43 ± 0.01^a#^	0.25 ± 0.01^a#^	0.60 ± 0.01^a^	0.09 ± 0.01^a^	0.83 ± 0.01^a^	5.66 ± 0.25^a^	8.32 ± 0.01^a^	1.54 ± 0.01^a^	950.47 ± 19.09^a^	49.48 ± 3.10^a^
47	0.41 ± 0.01^a#^	0.25 ± 0.01^a#^	0.58 ± 0.01^a^	0.04 ± 0.01^b^	0.97 ± 0.01^a^	4.01 ± 0.32^a^	9.72 ± 0.01^a^	1.46 ± 0.01^a^	960.41 ± 44.49^a^	44.31 ± 5.22^a^
48	0.43 ± 0.01^a#^	0.24 ± 0.01^a#^	0.60 ± 0.01^a^	0.05 ± 0.01^b^	0.89 ± 0.01^a^	6.27 ± 0.16^a^	8.94 ± 0.04^a^	1.40 ± 0.01^a^	980.42 ± 26.21^a^	42.30 ± 6.24^a^
49	0.42 ± 0.01^a#^	0.25 ± 0.01^a#^	0.57 ± 0.01^a^	0.09 ± 0.01^a^	0.87 ± 0.01^a^	4.15 ± 0.09^a^	8.65 ± 0.01^a^	1.61 ± 0.01^a^	904.15 ± 10.09^a^	46.80 ± 5.34^a^
50	0.42 ± 0.01^a#^	0.25 ± 0.01^a#^	0.58 ± 0.01^a^	0.10 ± 0.01^a^	0.83 ± 0.01^a^	4.01 ± 0.36^a^	8.28 ± 0.02^a^	1.54 ± 0.01^a^	907.46 ± 9.52^a^	46.42 ± 5.47^a^
*F/H*	2.77	2.86	2.98	10.08	3.52	13.73	3.55	3.07	2.89	3.65
*P*	0.20	0.18	0.15	0.04	0.13	0.04	0.13	0.14	0.16	0.13

The data with different superscript letters within the same column means significant difference, *P*<0.05, with the same superscript letter means no significant difference, *P*>0.05. The underlined data is *F* value; data without underline is *H* value

The results of Table 4 indicate that there have no statistically significant difference between the contents of lead, cadmium, chromium, arsenic, fluoride, copper, protein, phosphorus, potassium, and calcium in the rice including conventional rice, two-line hybrid rice, and three-line hybrid rice (*P* > 0.05).

**Table 4:** Test results of conventional rice, two-line hybrid rice, and three-line hybrid rice. ($\overline{X}\pm SD$)

	Limit standard (GB 2762-2012)	Rice	Conventional rice(*N* = 20) *n* = 120	Hybrid rice		*F/H*	
				Two-line hybrid rice (*N* = 15) *n* = 90	Three-line hybrid rice (*N* = 15) *n* = 90		*P*
Lead (mg/kg)	≤0.2	0.44 ± 0.02^#^	0.47 ± 0.02^a#^	0.43 ± 0.01^a#^	0.42 ± 0.02^a#^	1.87	0.32
Cadmium (mg/kg)	≤0.2	0.24 ± 0.02^#^	0.22 ± 0.01^a#^	0.24 ± 0.01^a#^	0.25 ± 0.01^a#^	5.18	0.12
Chromium (mg/kg)	≤1.0	0.57 ± 0.03	0.54 ± 0.02^a^	0.59 ± 0.02^a^	0.58 ± 0.02^a^	7.45	0.09
Inorganic arsenic (mg/kg)	≤0.2	0.10 ± 0.02	0.09 ± 0.01^a^	0.10 ± 0.03^a^	0.11 ± 0.02^a^	6.28	0.10
Fluorine (mg/kg)	Null	5.16 ± 1.35	5.42 ± 0.27^a^	4.93 ± 0.33^a^	5.03 ± 0.22^a^	4.04	0.18
Copper (mg/kg)	Null	0.86 ± 0.05	0.88 ± 0.06^a^	0.84 ± 0.05^a^	0.84 ± 0.03 ^a^	3.38	0.21
Crude protein (g/100 g)	Null	8.57 ± 0.52	8.81 ± 0.57^a^	8.43 ± 0.51^a^	8.40 ± 0.30^a^	3.40	0.20
Phosphorus (mg/kg)	Null	1.59 ± 0.12	1.61 ± 0.01^a^	1.56 ± 0.08^a^	1.60 ± 0.10^a^	4.40	0.17
Potassium (mg/kg)	Null	885.81 ± 201.38	1005.81 ± 203.89^a^	857.46 ± 19.75^a^	854.18 ± 14.38^a^	5.54	0.11
Calcium (mg/kg)	Null	53.78 ± 6.06	54.99 ± 5.09^a^	53.18 ± 6.78^a^	52.79 ± 6.26^a^	4.11	0.18

The data with different superscript letters within the same column means significant difference, *P*<0.05, with the same superscript letter means no significant difference, *P*>0.05. The underlined data is *F* value; data without underline is *H* value

### The BCFs of the lead, cadmium, chromium, arsenic, and copper from the soils to the rice

The results of the lead, cadmium, chromium, arsenic, and copper BCFs in the 50 different kinds of rice are shown in Table 5 below.

**Table 5:** Lead, cadmium, chromium, arsenic, and copper BCFs in 50 kinds of rice (*n* = 6, $\overline{X}\pm SD$)

Variety	Lead BCFs	Cadmium BCFs	Chromium BCFs	Arsenic BCFs	Copper BCFs
1	0.012 ± 0.00015^a^	0.461 ± 0.012^a^	0.014 ± 0.00009^a^	0.056 ± 0.0014^a^	0.093 ± 0.0036^a^
2	0.012 ± 0.00013^a^	0.464 ± 0.009^a^	0.015 ± 0.00007^a^	0.055 ± 0.0026^a^	0.081 ± 0.0051^a^
3	0.013 ± 0.00005^a^	0.455 ± 0.007^a^	0.016 ± 0.00007^a^	0.059 ± 0.0011^a^	0.085 ± 0.0026^a^
4	0.012 ± 0.00007^a^	0.460 ± 0.005^a^	0.015 ± 0.00010^a^	0.052 ± 0.0015^a^	0.084 ± 0.0021^a^
5	0.012 ± 0.00006^a^	0.459 ± 0.014^a^	0.015 ± 0.00004^a^	0.052 ± 0.0016^a^	0.097 ± 0.0028^a^
6	0.012 ± 0.00005^a^	0.459 ± 0.009^a^	0.015 ± 0.00004^a^	0.056 ± 0.0014^a^	0.095 ± 0.0014^a^
7	0.012 ± 0.00012^a^	0.457 ± 0.005^a^	0.015 ± 0.00009^a^	0.054 ± 0.0020^a^	0.094 ± 0.0053^a^
8	0.012 ± 0.00007^a^	0.454 ± 0.013^a^	0.014 ± 0.00002^a^	0.058 ± 0.0014^a^	0.093 ± 0.0085^a^
9	0.013 ± 0.00008^a^	0.454 ± 0.007^a^	0.015 ± 0.00004^a^	0.056 ± 0.0016^a^	0.087 ± 0.0029^a^
10	0.012 ± 0.00009^a^	0.457 ± 0.011^a^	0.014 ± 0.00009^a^	0.053 ± 0.0011^a^	0.075 ± 0.0045^a^
11	0.012 ± 0.00008^a^	0.462 ± 0.008^a^	0.015 ± 0.00005^a^	0.053 ± 0.0033^a^	0.076 ± 0.0013^a^
12	0.013 ± 0.00009^a^	0.464 ± 0.012^a^	0.015 ± 0.00007^a^	0.057 ± 0.0022^a^	0.076 ± 0.0037^a^
13	0.012 ± 0.00006^a^	0.454 ± 0.007^a^	0.015 ± 0.00008^a^	0.059 ± 0.0009^a^	0.084 ± 0.0027^a^
14	0.011 ± 0.00008^a^	0.457 ± 0.010^a^	0.014 ± 0.00004^a^	0.054 ± 0.0028^a^	0.079 ± 0.0054^a^
15	0.012 ± 0.00007^a^	0.459 ± 0.010^a^	0.015 ± 0.00009^a^	0.057 ± 0.0016^a^	0.085 ± 0.0013^a^
16	0.012 ± 0.00008^a^	0.454 ± 0.010^a^	0.016 ± 0.00006^a^	0.054 ± 0.0017^a^	0.108 ± 0.0034^a^
17	0.012 ± 0.00008^a^	0.462 ± 0.007^a^	0.014 ± 0.00005^a^	0.058 ± 0.0022^a^	0.096 ± 0.0040^a^
18	0.011 ± 0.0001^a^	0.459 ± 0.010^a^	0.015 ± 0.00004^a^	0.056 ± 0.0018^a^	0.090 ± 0.0022^a^
19	0.012 ± 0.0001^a^	0.535 ± 0.019^a^	0.015 ± 0.00006^a^	0.052 ± 0.0023^a^	0.089 ± 0.0012^a^
20	0.011 ± 0.00008^a^	0.535 ± 0.009^a^	0.016 ± 0.00011^a^	0.053 ± 0.0019^a^	0.034 ± 0.0017^b^
21	0.010 ± 0.00009^a^	0.536 ± 0.009^a^	0.017 ± 0.00011^a^	0.055 ± 0.0025^a^	0.079 ± 0.0012^a^
22	0.011 ± 0.00009^a^	0.556 ± 0.010^a^	0.016 ± 0.00008^a^	0.054 ± 0.0025^a^	0.088 ± 0.0010^a^
23	0.012 ± 0.00005^a^	0.562 ± 0.009^a^	0.014 ± 0.00004^a^	0.056 ± 0.0015^a^	0.078 ± 0.0013^a^
24	0.011 ± 0.00005^a^	0.532 ± 0.010^a^	0.015 ± 0.00008^a^	0.073 ± 0.0020^a^	0.088 ± 0.0038^a^
25	0.011 ± 0.00010^a^	0.561 ± 0.007^a^	0.017 ± 0.00006^a^	0.069 ± 0.0020^a^	0.086 ± 0.0054^a^
26	0.011 ± 0.00011^a^	0.553 ± 0.008^a^	0.017 ± 0.00010^a^	0.067 ± 0.0011^a^	0.087 ± 0.0012^a^
27	0.011 ± 0.00012^a^	0.544 ± 0.007^a^	0.016 ± 0.00011^a^	0.057 ± 0.0010^a^	0.083 ± 0.0021^a^
28	0.010 ± 0.00011^a^	0.563 ± 0.014^a^	0.017 ± 0.00008^a^	0.066 ± 0.0014^a^	0.082 ± 0.0029^a^
29	0.012 ± 0.00007^a^	0.553 ± 0.006^a^	0.016 ± 0.00006^a^	0.055 ± 0.0019^a^	0.099 ± 0.0028^a^
30	0.011 ± 0.00008^a^	0.573 ± 0.010^a^	0.017 ± 0.00007^a^	0.052 ± 0.0022^a^	0.084 ± 0.0083^a^
31	0.011 ± 0.00011^a^	0.547 ± 0.007^a^	0.017 ± 0.00004^a^	0.069 ± 0.0020^a^	0.079 ± 0.0034^a^
32	0.011 ± 0.00005^a^	0.574 ± 0.005^a^	0.016 ± 0.00004^a^	0.072 ± 0.0019^a^	0.100 ± 0.0030^a^
33	0.011 ± 0.00008^a^	0.552 ± 0.010^a^	0.017 ± 0.00004^a^	0.070 ± 0.0022^a^	0.083 ± 0.0038^a^
34	0.011 ± 0.00007^a^	0.557 ± 0.009^a^	0.017 ± 0.00006^a^	0.071 ± 0.0009^a^	0.081 ± 0.0035^a^
35	0.011 ± 0.00008^a^	0.556 ± 0.003^a^	0.018 ± 0.00002^a^	0.070 ± 0.0019^a^	0.077 ± 0.0028^a^
36	0.011 ± 0.00008^a^	0.547 ± 0.007^a^	0.017 ± 0.00007^a^	0.072 ± 0.0019^a^	0.080 ± 0.0026^a^
37	0.011 ± 0.00005^a^	0.550 ± 0.011^a^	0.017 ± 0.00004^a^	0.074 ± 0.0016^a^	0.082 ± 0.0025^a^
38	0.010 ± 0.00007^a^	0.553 ± 0.012^a^	0.016 ± 0.00005^a^	0.060 ± 0.0023^a^	0.078 ± 0.0016^a^
39	0.010 ± 0.00004^a^	0.548 ± 0.004^a^	0.016 ± 0.00009^a^	0.063 ± 0.0016^a^	0.079 ± 0.0033^a^
40	0.011 ± 0.00008^a^	0.539 ± 0.016^a^	0.016 ± 0.00008^a^	0.028 ± 0.0018^b^	0.073 ± 0.0054^a^
41	0.011 ± 0.00010^a^	0.560 ± 0.007^a^	0.017 ± 0.00005^a^	0.054 ± 0.0018^a^	0.070 ± 0.0017^a^
42	0.010 ± 0.00007^a^	0.552 ± 0.007^a^	0.017 ± 0.00003^a^	0.053 ± 0.0018^a^	0.136 ± 0.0039^c^
43	0.011 ± 0.00008^a^	0.541 ± 0.009^a^	0.017 ± 0.00008^a^	0.051 ± 0.0027^a^	0.122 ± 0.0013^c^
44	0.011 ± 0.00002^a^	0.534 ± 0.004^a^	0.017 ± 0.00008^a^	0.051 ± 0.0026^a^	0.072 ± 0.0023^a^
45	0.010 ± 0.00009^a^	0.549 ± 0.017^a^	0.016 ± 0.00005^a^	0.053 ± 0.0014^a^	0.088 ± 0.0048^a^
46	0.011 ± 0.00005^a^	0.558 ± 0.011^a^	0.017 ± 0.00009^a^	0.054 ± 0.0016^a^	0.078 ± 0.0052^a^
47	0.010 ± 0.00006^a^	0.548 ± 0.013^a^	0.016 ± 0.00007^a^	0.024 ± 0.0016^b^	0.085 ± 0.0005^a^
48	0.011 ± 0.00007^a^	0.539 ± 0.009^a^	0.017 ± 0.00007^a^	0.027 ± 0.0012^b^	0.087 ± 0.0064^a^
49	0.011 ± 0.00006^a^	0.553 ± 0.014^a^	0.016 ± 0.00006^a^	0.060 ± 0.0028^a^	0.087 ± 0.0013^a^
50	0.011 ± 0.00037^a^	0.570 ± 0.009^a^	0.017 ± 0.00007^a^	0.054 ± 0.0017^a^	0.085 ± 0.0012^a^
*F/H*	1.98	2.90	2.63	11.01	10.23
*P*	0.25	0.16	0.20	0.04	0.04

The data with different superscript letters within the same column means significant difference, *P*<0.05, with the same superscript letter means no significant difference, *P*>0.05. The underlined data is *F* value; data without underline is *H* value

Table 5 shows the lead, cadmium, and chromium BCFs in the rice have no statistically significant differences among the various species (*P* > 0.05). Compared with other species of rice, the arsenic BCFs of no. 40, 47, 48, and the copper BCFs of the no. 20 are significantly lower (*P* < 0.05), and the copper BCFs of the no. 42 and 43 varieties are higher (*P* < 0.05).

Given the exceeded lead and cadmium BCFs in the 50 different kinds of rice, which do not have a statistically significant difference among all the varieties, we will analyze the data of rice in the perspective of the conventional rice, two-line hybrid rice, and the three-line hybrid rice. Table 6 shows the test results of the BCFs of the traces of lead, cadmium, chromium, arsenic, and copper found in the traditional rice, two-line hybrid rice, and three-line hybrid rice samples.

**Table 6:** BCFs of lead, cadmium, chromium, arsenic, and copper in traditional rice, two-line hybrid rice, and three-line hybrid rice ($\overline{X}\pm SD$)

Variety	Lead BCFs	Cadmium BCFs	Chromium BCFs	Arsenic BCFs	Copper BCFs
Conventional rice	0.012 ± 0.0004^a^	0.459 ± 0.010^a^	0.015 ± 0.0005^a^	0.056 ± 0.0046^a^	0.090 ± 0.0057^a^
Two-line hybrid rice	0.011 ± 0.0003^a^	0.547 ± 0.012^a^	0.017 ± 0.0005^a^	0.058 ± 0.0042^a^	0.084 ± 0.0042^a^
Three-line hybrid rice	0.011 ± 0.0004^a^	0.552 ± 0.016^a^	0.017 ± 0.0005^a^	0.062 ± 0.0034^a^	0.087 ± 0.0040^a^
*F/H*	2.94	3.29	3.17	2.54	0.67
*P*	0.09	0.08	0.08	0.09	0.52

The data with different superscript letters within the same column means significant difference, *P*<0.05, with the same superscript letter means no significant difference, *P*>0.05. The underlined data is *F* value; data without underline is *H* value

Table 6 shows lead BCFs, chromium BCFs, arsenic BCFs, cadmium BCFs, and copper BCFs of conventional rice, two-line and three-line hybrid rice have no statistically significant differences (*P* > 0.05).

## Discussion and analysis

Heavy metal pollution of soil has become a global environmental issue (Salvatore et al. 2009). Due to the growth characteristics and the genetic characteristic differences among the different rice crops, there are significant variances in the amounts of heavy metals the different rice types have absorbed, as well as there being differences in bioconcentration process and characteristics among the samples. Heavy metal pollution is caused by several factors such as, the naturally occurring content of heavy metals in soil, physicochemical properties, the existing forms in different soil and plant types, the growth cycle, the quality of the atmosphere of the planting environment, irrigation water and fertilizer. Specifically, soil, sewage irrigation, fertilizer, and air dust suppression are the main factors, which influence the content of heavy metals in crops. When the heavy metal content of crops exceeds the maximum allowable concentration, it will endanger the safety of people and animals that consume it, having a dire affect on the entire food chain (Handique et al. 2009; Beyersmann et al. 2008). Therefore, this research aims to investigate the content of heavy metals in rice and BCFs, known as the bioconcentration situation of heavy metals, in 50 separate kinds of rice under the same environmental conditions, and to provide the scientific basis for further study.

As previous research has shown, the mean value of lead content in soil is 27.93 mg/kg Shi, and the lead concentration in a single rice grain ranges from 0.05 to 0.74 mg/kg with the mean value of 0.27 mg/kg, which is 1.35 times the permitted value (0.2 mg/kg), and the over standard rate was up to 58.6%. Since the overall accumulation of lead is severe, it is necessary to focus on monitoring the lead content in rice grains (Wang and Gong 1996).

The analytical results, based on the testing of lead, cadmium, and arsenic content in a test site, displayed that there are excessive levels of lead, cadmium, and arsenic contents in rice and they are over the limited standards set in the test site, with lead being the main factor causing heavy metal pollution in rice (Li et al. 2015). Our results indicate that the lead content of the soil samples collected is 48.7 mg/kg, leading to 94.3% of the samples beyond the standards set by environmental and governmental agencies (Yang et al. 2013); when the lead content of rice is 58 mg/kg, then the lead content of rice has increased by around 12.4 mg/kg(Li et al. 2003). Thus, our research results demonstrate that the test values of irrigation water meet the farmland irrigation water standards. The lead content of the paddy mud sample is 39.56 ± 2.16 mg/kg, which is far below the pH value of 6.5–7.5, set by the national edible agricultural origin environment standards, where the evaluation standard of lead content is ≤80 mg/kg. Moreover, the lead content in 50 species of rice is 0.41 ± 0.01~0.49 ± 0.01 mg/kg, which exceeds the limit of rice pollutants as set (by Food and Drug Administration). The varieties of the different kinds of rice have no significant statistical meaning (*P* > 0.05). Hence, consistent with the above research results, it is necessary to investigate and revise lead limit standards of environmental quality of edible agricultural products, based on the consideration about the strong adsorption capacity of lead in rice.

Metal pollutants in the soil activity directly affect rice’s biological effectiveness. The pollution of rice is caused by cadmium residue having an especially high exchange transference rate, strong mobility, and high bio-availability (Jung et al. 1997; Kashem et al. 2001; Liu et al. 2005; Li et al. 2008; Wang et al. 2012). The cadmium detected in the rice samples was significantly associated with the cadmium levels detected in the planting soil (*P* < 0.01) (Feng et al. 2011). When the cadmium content in soil is less than 0.3 mg/kg, the exceeded cadmium content in rice increases by around 43.3%. When the cadmium content in the soil is greater than 1.0, the exceeded cadmium content in rice is 3.7 times that of the limited standards set by Food and Drug Administration. When the cadmium content in soil is between 0.3 and 1.0, the exceeded cadmium content in rice is twice the amount of the limited standard. When the cadmium content in soil is less than 0.3 g, the exceeded cadmium content in the rice is 1.4 times that of the limited standard. These results indicate then that when the cadmium content of soil is higher, the same will happen to the cadmium content of the rice. Thus, the cadmium content in soil is an important factor to determine the cadmium content of the rice grown in that soil (Peng et al. 2013). Therefore, our research concludes that the amount of cadmium content in rice field soil, which exceeds the environmental quality assessment standard of edible agricultural products, is 0.07 mg/kg. The cadmium content of 50 types of rice is 0.22 ± 0.01~0.25 ± 0.01 mg/kg, which is slightly higher than the limits set by Food and Drug Administration, and which shows no statistically significant difference between the varieties of rice (*P* > 0.05). This result is not fully consistent with the above research results, when taking into account the factor that whether or not the quality of irrigation water in test sites complies with the quality standards of irrigation water.

The BCFs, as the ratio between contaminants in the object and the corresponding pollution levels in the soil, are used to evaluate rice’s bioconcentration ability to absorb heavy metalin quantities from the soil. The larger the amount of BCFs, the more heavy metals the rice is capable of absorbing and accumulating from the soil. A research reported by Li stated that when the lead, cadmium, arsenic, and copper contents are respectively 46.53, 0.42, 11.17, and 38.74 mg/kg in a paddy field, the BCFs of lead, cadmium, arsenic, and copper are respectively 0.0012, 0.4782, 0.0200, and 0.2232 in the rice (Li et al. 2003b). The BCFs of Cd in rice could reach up to 0.47 when the cadmium content of soil is more than 0.3 g (Song et al. 2000). When soil pollution is comparatively serious, it will increase the amount of BCFs of heavy metals found in brown rice grown in that soil (Zhang et al. 2012). This research illustrates, then, that the bioconcentration ability of lead, cadmium, and chromium, as found in 50 different kinds of rice, has no difference. The bioconcentration ability of the arsenic found in the three rice varieties and one rice variety, for copper, is relatively lower than the bioconcentration ability of other heavy metals, while the bioconcentration ability for copper in the two rice varieties is relatively higher. The bioconcentration ability for lead, cadmium, chromium, arsenic, and copper, among the conventional rice, the two-line hybrid rice and the three-line hybrid rice, has no significant statistical difference between them. These results indicate that the different varieties of rice have no relation to the bioconcentration ability of heavy metals for rice from the same soil background.

## Conclusions

In summary, this study investigated the lead and cadmium contents of 50 kinds of rice which all exceed the contaminant limit standards, as set by Food and Drug Administration. The cadmium content in the soil we analyzed was far beyond the normal evaluation standard of 0.07 mg/kg. The cadmium contents found in 50 different kinds of rice exceeds the contaminant limit standard 0.04 mg/kg. However, the lead content found in the soil was within the evaluation standard, but the lead contents of the 50 different kinds of rice were beyond the set contaminant limit standard of 0.24 mg/kg. Furthermore, the bioconcentration ability of lead, cadmium, and chromium, in 50 different kinds of rice, had no difference. For the bioconcentration of lead, cadmium, chromium, arsenic, and copper, there was no significant difference between conventional, two-line hybrid rice, and three-line hybrid rice. Therefore, we conclude that rice has a strong adsorption capacity for lead, which means it is necessary to conduct further research on control measures that could reduce heavy metal pollution in rice. Additionally, it is also important to investigate the lead limit standards of environmental quality of edible agricultural products, which will provide guidance for heavy metal pollution under different environmental conditions.
